# Stable Density and Dynamics of Dendritic Spines of Cortical Neurons Across the Estrous Cycle While Expressing Differential Levels of Sensory-Evoked Plasticity

**DOI:** 10.3389/fnmol.2018.00083

**Published:** 2018-03-16

**Authors:** Bailin H. Alexander, Heather M. Barnes, Emma Trimmer, Andrew M. Davidson, Benard O. Ogola, Sarah H. Lindsey, Ricardo Mostany

**Affiliations:** ^1^Department of Pharmacology, Tulane University School of Medicine, Tulane University, New Orleans, LA, United States; ^2^Neuroscience Program, Brain Institute, Tulane University, New Orleans, LA, United States; ^3^Department of Cell and Molecular Biology, Tulane University, New Orleans, LA, United States; ^4^Brain Institute, Tulane University, New Orleans, LA, United States

**Keywords:** synaptic plasticity, estrous cycle, two-photon imaging, pyramidal neurons, female, estrogens, dendritic spines

## Abstract

Periodic oscillations of gonadal hormone levels during the estrous cycle exert effects on the female brain, impacting cognition and behavior. While previous research suggests that changes in hormone levels across the cycle affect dendritic spine dynamics in the hippocampus, little is known about the effects on cortical dendritic spines and previous studies showed contradictory results. In this *in vivo* imaging study, we investigated the impact of the estrous cycle on the density and dynamics of dendritic spines of pyramidal neurons in the primary somatosensory cortex of mice. We also examined if the induction of synaptic plasticity during proestrus, estrus, and metestrus/diestrus had differential effects on the degree of remodeling of synapses in this brain area. We used chronic two-photon excitation (2PE) microscopy during steady-state conditions and after evoking synaptic plasticity by whisker stimulation at the different stages of the cycle. We imaged apical dendritic tufts of layer 5 pyramidal neurons of naturally cycling virgin young female mice. Spine density, turnover rate (TOR), survival fraction, morphology, and volume of mushroom spines remained unaltered across the estrous cycle, and the values of these parameters were comparable with those of young male mice. However, while whisker stimulation of female mice during proestrus and estrus resulted in increases in the TOR of spines (74.2 ± 14.9% and 75.1 ± 12.7% vs. baseline, respectively), sensory-evoked plasticity was significantly lower during metestrus/diestrus (32.3 ± 12.8%). In males, whisker stimulation produced 46.5 ± 20% increase in TOR compared with baseline—not significantly different from female mice at any stage of the cycle. These results indicate that, while steady-state density and dynamics of dendritic spines of layer 5 pyramidal neurons in the primary somatosensory cortex of female mice are constant during the estrous cycle, the susceptibility of these neurons to sensory-evoked structural plasticity may be dependent on the stage of the cycle. Since dendritic spines are more plastic during proestrus and estrus than during metestrus/diestrus, certain stages of the cycle could be more suitable for forms of memory requiring *de novo* formation and elimination of spines and other stages for forms of memory where retention and/or repurposing of already existing synaptic connections is more pertinent.

## Introduction

Over the past 40 years it has been well established that gonadal hormones exert influence on brain structure and function in both the developing and the adult brain (Luine and Rodriguez, 1994; McEwen and Alves, 1999; Brinton, 2009; Srivastava et al., 2013). It is also well known that brain functions depend on the maintenance of synaptic contacts, as well as on the ability to modify, eliminate, and create new synapses between neurons to establish adaptive connections in a changing environment (Holtmaat and Svoboda, 2009). This degree of adaptability is defined as synaptic plasticity, and it is paramount for any type of learning and form of memory. Dendritic spines are essential components of cortical circuits, functioning as the main postsynaptic structure receiving excitatory inputs in pyramidal neurons in the neocortex and as the anatomical substrate for memory storage (Yuste and Bonhoeffer, 2001). These neuronal structures have been used as a proxy of the computing capabilities of the cortical circuitry and can undergo rapid and extensive changes during learning (Xu et al., 2009; Lai et al., 2012; Moczulska et al., 2013; Kuhlman et al., 2014) and after sensory manipulations (Zuo et al., 2005; Miquelajauregui et al., 2015) or brain injury (Mostany et al., 2010). Several studies have reported changes in spine density in the hippocampus at different stages of the estrous cycle, with higher levels of estrogen during proestrus corresponding to higher densities of dendritic spines (Woolley et al., 1990; Woolley and McEwen, 1992; Kato et al., 2013). However, results from studies in the cerebral cortex are more arguable, with studies reporting higher density of dendritic spines during proestrus (Chen et al., 2009) and studies reporting no differences in density in relation to the stage of the estrous cycle (Markham and Juraska, 2002; Prange-Kiel et al., 2008).

Regardless of the results, these studies used fixed tissues, a methodology that does not allow tracking spine density and dynamics longitudinally, and no *in vivo* studies are available on the effects of fluctuating hormone levels on cortical plasticity. The advent of *in vivo* two-photon excitation (2PE) imaging (Denk et al., 1990; Svoboda and Yasuda, 2006) has allowed the precise, longitudinal examination of dendritic spines in the same animal. Several 2PE imaging studies have shown that sensory manipulations affect the dynamics of dendritic spines of different cortical areas in mice, showing increases in the turnover ratios of dendritic spines after monocular deprivation (Hofer et al., 2009) or whisker trimming (Trachtenberg et al., 2002). However, it is unknown whether these changes in the dynamics of dendritic spines in response to a sensory-dependent manipulation are similar in magnitude or duration in females across the estrous cycle.

The goal of the present *in vivo* study was to characterize the changes in dendritic spine density and dynamics of cortical pyramidal neurons of naturally cycling female mice and of male mice to better understand whether the mechanisms regulating memory and learning are enhanced or weakened at specific stages of the cycle and whether these mechanisms are different between female and male mice. These are important pieces of information that are still missing in the neurophysiology of the female brain. Based on the results from previous studies, we wanted to test the hypothesis that dendritic spine density of L5 pyramidal neurons varies during the estrous cycle as a consequence of the oscillating levels of endogenous estrogen. We also wanted to test if synaptic plasticity was differentially expressed in L5 pyramidal neurons across the estrous cycle. Finally, we wanted to test the hypothesis that both, dendritic spine density and dynamics, are different between female and male due to the effect of endogenous hormones associated with the estrous cycle in female mice. We found that dendritic spine density is constant across the estrous cycle in females and similar in magnitude to the density found in males. We also found that in steady-state conditions, the dynamics of dendritic spines are not affected by the stage of the cycle and are comparable to the dynamics observed in males. Finally, we found that the response of these neurons to sensory-evoked structural plasticity is dependent on the stage of the cycle.

## Materials and Methods

### Animals

We used GFP-M transgenic mice expressing GFP under the Thy-1 promoter (The Jackson Laboratory; IMSR Cat# JAX:007788, RRID:IMSR_JAX:007788). These mice express GFP in sparse subsets of projection pyramidal track-type layer 5 (L5) pyramidal neurons in the cortex (Feng et al., 2000). All animals were virgin and group caged. Food and water was available *ad libitum* and all cages were kept in a 12-h light/dark cycle. The study was carried out in accordance with the recommendations of the NIH Office of Laboratory Animal Welfare’s *Public Health Service Policy on Humane Care and Use of Laboratory Animals* and *Guide for the Care and Use of Laboratory Animals* and all the procedures described were approved by the Institutional Animal Care and Use Committee of Tulane University.

### Droplet Digital PCR

Four GFP-M female mice (3.7 ± 0.2 months of age) were decapitated and immediately dissected on ice, and the primary somatosensory cortex was removed from both hemispheres. Tissues were immediately immerged in RNA*later* Stabilization Solution (Invitrogen Cat# AM7020) and kept at 4°C for 24 h before freezing. Tissues from the left hemisphere were used for droplet digital PCR (ddPCR). RNA was isolated using the RNeasy mini kit (Qiagen). Purity and concentration of RNA was determined using the nanodrop before being subjected to ddPCR as previously described (Liu et al., 2017). Briefly, approximately 40 ng of RNA was combined with Taqman primers and probes as well as Mastermix (Bio-Rad) to create a 20 μl working reaction mixture (Hindson et al., 2011). The following PrimePCR Primers were used: G protein-coupled estrogen receptor 1 (ER) 1 (GPER/GPR30; Bio-Rad Unique Assay ID: dMmuCPE5103030), ER alpha (ERα, ESR1; Bio-Rad Unique Assay ID: dMmuCPE5092741), ER beta (ERβ, ESR2; Bio-Rad Unique Assay ID: dMmuCPE5092742), and aromatase/CYP19A1 (Bio-Rad Unique Assay ID: dMmuCPE5097863). Each working reaction mixture was then fractionated into approximately 20,000 individual 1 nl droplets by oil emulsion microfluidics. During thermal cycling, each droplet comprises an individual PCR reaction. If the target sequence is present, a fluorescent reporter probe is released. Droplets were analyzed via the QX200 droplet reader (BioRad), which samples and singularizes each reaction mixture, flowing droplets at a rate of 1000 droplets/s past a two-color fluorescence detector. The ratio of positive to negative droplets allows the system to compute the concentration of a target sequence via Poisson distribution statistics.

### Western Blot Detection of Classic Nuclear ERs

The tissues from the right hemispheres from the same animals used for the ddPCR were used for immunoblotting assays. Samples were homogenized in NP40 Cell Lysis Buffer (ThermoFisher Cat# FNN0021). Following a 10-min centrifugation at 10,000 rpm, protein concentration was quantified using Pierce BCA Protein Assay Kit (ThermoFisher Cat# 23225). For gel electrophoresis, 100 μg of protein was loaded into 10% SDS-polyacrylamide gels (Invitrogen Cat# NP0315BOX) and was resolved at 150 V for approximately 90 min. Next, the gels were blotted to nitrocellulose membrane (Bio-Rad Cat# 1620115) via iBlot System (Invitrogen). Blots were washed once with DI water then incubated in 5% milk solution at room temperature for 2 h. They were subsequently incubated overnight at 4°C with the primary antibody (1:1000 in Odyssey Blocking Buffer; Li-Cor Biosciences Cat# 927-40100). Next, the blots were washed with TBST (3 × 10 min) then incubated for 2 h at room temperature with the secondary antibody (1:1000 in 5% milk in TBST solution). Immunoblotting was done using anti-ER α (Santa Cruz Biotechnology Cat# sc-71064, RRID:AB_1122667) and anti-ER β (Abcam Cat# ab288, RRID:AB_303379), each paired with a secondary anti-mouse antibody (Santa Cruz Biotechnology Cat# sc-516102, RRID:AB_2687626) conjugated to horseradish peroxidase. Immunoreactive bands were visualized using SuperSignal West Pico Kit (ThermoFisher Cat# 34580).

### Cranial Window Surgery for *in Vivo* Imaging

**Figure 1:**
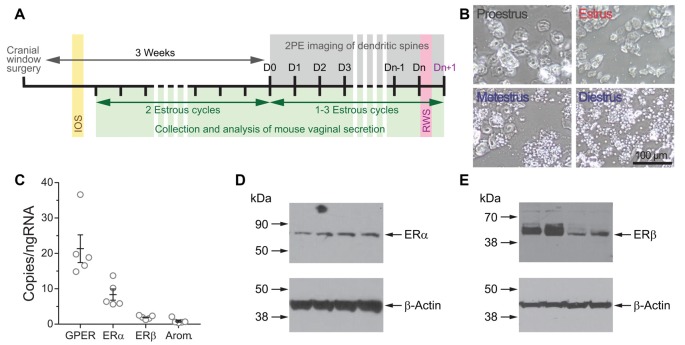
**(A)** Experimental design for time-lapse two-photon imaging of dendrites during the estrous cycle. **(B)** Representative photomicrographs of vaginal cytology from mice at the different stages of the estrous cycle. **(C)** Copies per ng of RNA of transcripts for G protein-coupled estrogen receptor (GPER), estrogen receptor α (ERα), ERβ, and aromatase in the somatosensory cortex of Thy1 GFP-M female mice determined by droplet digital PCR (ddPCR). **(D,E)** The expression of ERα **(D)** and ERβ **(E)** in the somatosensory cortex of Thy1 GFP-M female mice was confirmed by Western blot.

Chronic glass-covered cranial windows were implanted as described previously (Mostany and Portera-Cailliau, 2008; Holtmaat et al., 2009) at least 3 weeks before the beginning of the *in vivo* imaging of dendritic spines (Figure 1A). The average age at the time of entering the experiment was 3.5 ± 0.9 months (mean ± SD; range 2.1–4.8 months) and 3.1 ± 0.7 months (range 2.3–4.4 months) for the female and male mice, respectively. Briefly, mice were anesthetized (isoflurane, 5% for induction, 1.5% for maintenance via nose cone) and placed on a stereotaxic frame. Dexamethasone (0.2 mg/kg; MWI/VetOne) and carprofen (Rimadyl^®^ 5 mg/kg; Zoetis, Inc.) were administered subcutaneously to reduce brain edema and local tissue inflammation. A 4 mm-diameter craniotomy was performed with a pneumatic dental drill over the primary somatosensory cortex, 3 mm lateral to the midline and 1.95 mm caudal to Bregma. A round glass coverslip (5 mm #1; Electron Microscopy Sciences) was gently laid over the dura mater, covering the exposed brain and part of the skull and glued to the latter with cyanoacrylate-based glue. A layer of dental acrylic (Lang Dental Mfg. Co., Inc.) was then applied throughout the skull surface and up to the edges of the coverslip. A titanium bar (9.5 × 3.2 × 1.1 mm) was embedded in the dental acrylic to secure the mouse onto the stage of the microscope for imaging.

### Vaginal Cytology

Female mouse estrous cycles were tracked beginning 2 weeks post-cranial window surgery using a wet smear technique as previously described (Caligioni, 2009). Briefly, a standard 10 μl pipette loaded with 10 μl of 0.9% saline solution was used to flush the vaginal cavity at approximately the same time each day, 10 am ± 1 h. The samples were scored on an inverted light microscope (Fisher Scientific Company LLC) and assigned to a stage of the estrous cycle (Figure 1B). Smears that contained primarily nucleated epithelial cells and absence or very low numbers of leukocytes were classified as proestrus (P). Smears that contained predominantly cornified squamosal cells were classified as estrus (E), and those containing abundant numbers of cornified cells and/or significant amounts of leukocytes were classified as metestrus/diestrus (M/D; Caligioni, 2009). All female mice were followed through two complete cycles before imaging was started to ensure the animal was cycling regularly. Females that showed irregular estrous cycles (i.e., stayed 50% of time in one stage, skipped stages, or average cycle length was ≥7 days) were excluded from the study. Once the females entered the *in vivo* imaging regime, the lavages were performed immediately after the *in vivo* imaging session and while the female was still anesthetized.

### Intrinsic Optical Signal Imaging

The precise location of the primary somatosensory cortex barrel field (S1BF) was mapped through the cranial window preparation using intrinsic optical signal (IOS) imaging, as previously described (Johnston et al., 2013). Briefly, the mice were anesthetized with isoflurane (0.75%–1.0% via nose cone) and secured to the IOS rig using the titanium head bar. LED array light sources (Thorlabs) of green (525 nm) and red (630 nm) were used to visualize the vasculature and for the IOS imaging, respectively. Imaging was performed at 30 frames per second using a Pantera 1M60 camera (Dalsa) and custom routines written in MATLAB (MathWorks; RRID:SCR_001622). The camera was focused to approximately 350 μm below the dura surface using a tandem of objective lenses (135 mm and 50 mm) arranged in a front-to-front configuration. The vibrissae contralateral to the cranial window were bundled and fixed with dental wax to a glass microelectrode coupled to a piezo bender actuator (Physik Instrumente). Imaging sessions consisted of 30 trials, taken 20 s apart, of mechanical stimulation in the rostro-caudal direction for 1.5 s at 10 Hz. Frames 0.9 s before onset of stimulation (baseline) and 1.5 s after stimulation (response) were collected. Stimulated cortical areas (barrel cortex) were identified by dividing the response signal by the averaged baseline signal (ΔR/R) for every trial and then summing all trials.

### High Resolution *in Vivo* Two-Photon Imaging

Imaging of dendritic spines was done under isoflurane anesthesia (1%–1.5%) with a custom-built two-photon microscope, using a Ti:Sapphire laser (Chameleon Ultra II; Coherent Inc.) tuned to 910 nm, a 40× 0.8 NA water immersion objective (Olympus), and ScanImage 3.8 software (RRID:SCR_014307; Pologruto et al., 2003) written in MATLAB (MathWorks; RRID:SCR_001622). Mice were secured to the microscope using the titanium head bar. Guided by the maps obtained with the IOS imaging, we scanned the S1BF area searching for L5 pyramidal neurons with apical dendritic tufts clearly identifiable and not obscured by large blood vessels. On the first day of imaging, low magnification image stacks (512 × 512 pixels, 0.72 μm/pixel, 5 μm *z* steps) were collected down to the soma, at least 450 μm from the pia, to ensure all cells studied were L5 pyramidal neurons. The apical dendritic tufts of L5 pyramidal neurons were imaged every 24 h (Figure 1A), ranging between 8 am and 12 pm for all the mice imaged. High magnification images (512 × 512 pixels, 0.152 μm/pixel, 1.5 μm *z* steps) of dendritic segments in L1 (within the first 100 μm from the pia mater) were collected on each day of imaging for the analysis of dendritic spines. After female mice demonstrated that they were having regular menstrual cycles, they were imaged daily for 1–3 additional menstrual cycles (average: 2.1 ± 0.8 cycles/female) for an average of 12.4 ± 2.5 days for assessment of steady-state dynamics of dendritic spines (Figure 1A). Imaging of male mice began 3 weeks post-cranial window surgery, and they were imaged consecutively for 7 days for assessment of steady-state dynamics of dendritic spines.

### Sensory-Evoked Synaptic Plasticity

Once the female mice completed the *in vivo* imaging regime for the study of steady-state density and dynamics of dendritic spines across the estrous cycle, they were randomly assigned to one of the experimental groups (P_St_, E_St_, or M/D_St_) based on the stage of the cycle at which the female mouse was going to experience the induction of synaptic plasticity. Daily imaging continued until the assigned estrous stage was detected after the two-photon imaging session. At that moment we induced sensory-evoked plasticity by rhythmic sensory whisker stimulation (RWS; Figure 1A) following a stimulation protocol previously described to induce long term plasticity (Mégevand et al., 2009; Gambino et al., 2014). Briefly, at the conclusion of the 2PE imaging session, and after assessing the stage cycle, mice were lightly anesthetized (0.75%–1% isoflurane) and attached to the rig using the titanium head bar. The vibrissae contralateral to the cranial window were bundled and fixed with dental wax to a glass microelectrode coupled to a piezo bender actuator which continuously vibrated the whiskers with rostro-caudal deflections (2 mm) at a rate of 8 Hz for 10 min. Male mice underwent whisker stimulation immediately after the last imaging session of the baseline period (day 7). All mice underwent one final imaging session 24 h after the stimulation.

### Analysis

Spine density and dynamics from 2PE images were determined using spine analysis software written in MATLAB (kindly provided by Drs. T. O’Connor and K. Svoboda, Janelia Campus). All visible spines were scored, including those on the z-axis which clearly protruded beyond the noise of the dendritic shaft. Scoring of dendritic spines was done blind to both the estrous cycle and sex. Within each cycle, the data obtained for a given stage was averaged if that particular estrous stage lasted for more than 1 day. Values for each of the stages from consecutive cycles were averaged. We analyzed dendritic spines from *n* = 15 cells (*n* = 10 female mice) and *n* = 10 cells (*n* = 6 male mice) and tracked a total of 5890 distinct dendritic spines over 6–18 imaging sessions. For display purposes only, best projections of the dendritic segments were obtained, where the best focal plane is identified and overlaid in Adobe Photoshop CC (Adobe Systems Inc.; RRID:SCR_014199), preserving all the elements in the segment, and a median filter (radius of 1) was applied. The fold-change in spine density was defined as the density calculated the day after the stimulation divided by the average spine density computed during the steady-state imaging regime. We defined turnover rate (TOR) of dendritic spines as: combined number of gained and lost spines divided by two-times the total number of spines ([#gained spines + #lost spines]/2 × total number of spines). The fold-change in TOR was defined as the TOR computed the day after the stimulation divided by the average TOR computed during the steady-state imaging regime. New persistent spines were defined as newly-formed spines that were still present 24 h after their initial formation. The survival function over a period of 6 days of dendritic spines present at a given day was obtained by fitting the survival fraction plots to a single exponential decay curve. Survival fraction and half-life were calculated as follows: survival fraction = plateau + unstable fraction X e^−t/τ^, where *t* is the time (days) and τ (tau) is the time constant. Rate constant (K) = 1/τ. Volumetric estimations of dendritic spines and sorting of dendritic spine subtypes were completed using custom written routines in MATLAB and ImageJ (RRID:SCR_003070)^1^ as previously described (Mostany et al., 2013). Briefly, dendritic spine volumes were calculated by total integrated brightness (TIB) and normalized using the mean TIB of the adjacent dendritic shaft. Dendritic spines were classified (stubby, thin or mushroom) using a semiautomated unsupervised method. The examiner drew a line manually from the center of the dendritic shaft to the tip of the spine and the pixel intensity for the profile was obtained. A custom written MATLAB routine used the brightness intensity data to determine the spine type based on several criteria including presence or absence of a spine head and overall spine length. Volumetric estimations were limited to stable mushroom spines across three consecutive stages of the estrous cycle in female mice and in male mice. All cells that were used for volumetric analysis were also analyzed for dendritic spine subtypes (*n* = 15 cells in *n* = 10 female mice). An average of 19 randomly selected stable mushroom spines (range 16–23) were tracked in each cell across the three estrous cycle stages and in male mice (285 individual mushroom spines) for volumetric comparison. For dendritic spine sorting for the population study, an average of 460 dendritic spines per stage of the cycle (92 dendritic spines/mouse) were scored. For longitudinal analyses of dendritic spine morphology, a total of 876 spines were scored.

### Statistics

Statistical differences between groups were calculated with repeated measures one-way or two-way ANOVA followed by a Tukey’s multiple comparison test when applicable. A Student’s *t* test (unpaired or paired when appropriate) was used to compare single parameters between two groups. The extra sum-of-squares *F* test was used to compare the best-fit values for the survival function parameters, i.e., plateau and rate constant K. Differences in proportions between groups was computed using a Chi-square test. All statistical analyses were performed with GraphPad Prism (GraphPad Software; RRID:SCR_002798). All data are presented as the mean ± SEM, unless otherwise stated. Significance was set at *p* < 0.05. In the figures, *p < 0.05, **p < 0.01, and ***p < 0.001.

## Results

### The Primary Somatosensory Cortex Barrel Field Is a Putative Estrogen-Sensitive Area

While several studies have previously shown the presence of ERα and ERβ receptors in total cerebral cortex lysates of mice (Sharma and Thakur, 2006), in the somatosensory cortex (Mitra et al., 2003), as well as their mRNAs from mouse total cerebral cortex (Thakur and Sharma, 2007) and mouse primary visual cortex (Jeong et al., 2011), there is no data about the expression of these receptors in the somatosensory cortex of Thy1 GFP-M mice. The expression of aromatase mRNA has been detected in the visual cortex (Jeong et al., 2011) and in the cingulate and secondary motor cortex (Stanić et al., 2014) of mice. However, the expression of this enzyme has not been reported in the Thy1 GFP-M mice used in this study. The expression of RNA transcripts for ERs ERα and ERβ, GPER, and aromatase was confirmed by ddPCR (Figure 1C). Western blot assays confirmed the expression of the two classic ERs ERα and ERβ (Figures 1D,E). These results indicate that the primary somatosensory cortex of the transgenic Thy1 GFP-M mice is a putative estrogen-sensitive area.

### Density, Turnover, Survival Fraction, Morphology, and Volume of Dendritic Spines in the Somatosensory Cortex of Adult Female Mice Does Not Differ Between Stages of the Estrous Cycle

**Figure 2:**
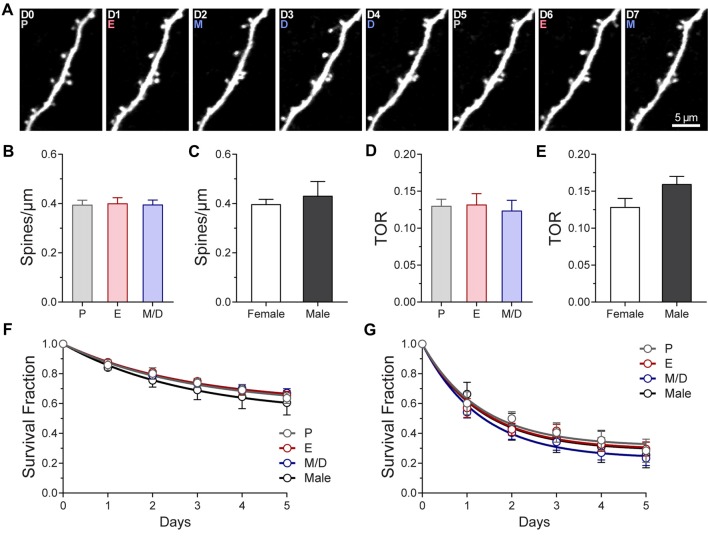
Density and turnover of dendritic spines of L5 pyramidal neurons in primary somatosensory cortex barrel field (S1BF) across the estrous cycle. **(A)** Representative high-resolution two-photon microscopy images acquired *in vivo* of an apical dendritic segment from a L5 pyramidal neuron across two estrous cycles. All the images are best projections (3–6 slices, 1.5 μm apart). The day of imaging and the stage of the cycle are shown in the upper left corner. **(B)** Density of dendritic spines in S1BF at the different stages of the estrous cycle.** (C)** Average density of dendritic spines in female and male mice. **(D)** Turnover rate (TOR) of dendritic spines in S1BF at the different stages of the estrous cycle. **(E)** Average TOR of dendritic spines in female and male mice. **(F,G)** Survival fraction of all the spines present **(F)** or new persistent spines formed **(G)** at different stages of the estrous cycle and in male mice. P, Proestrus; E, Estrus; M/D, Metestrus/Diestrus.

We used high-resolution *in vivo* two-photon imaging of layer (L) 5 pyramidal neurons through a cranial window preparation to monitor the density, dynamics, size, and morphology of individual dendritic spines in the primary somatosensory cortex barrel field (S1BF) of GFP-M female mice across the estrous cycle. To characterize the steady-state density and dynamics of dendritic spines, imaging sessions were performed at 24-h intervals through the estrous cycle (Figures 1A, 2A). To further assess whether sex-differences in density and dynamics of dendritic spines exist in S1BF, male mice were imaged daily for 7 days to obtain the same metrics. No differences in dendritic spine density (# spines/μm) were found between any stages of the estrous cycle in regularly cycling females (P: 0.39 ± 0.02; E: 0.40 ± 0.02, M/D: 0.40 ± 0.02 spines/μm; *p* = 0.57, repeated measures one-way ANOVA; Figure 2B). Additionally, the average dendritic spine density across all the stages in females did not differ from the average density observed in males (0.40 ± 0.02 spines/μm in females vs. 0.43 ± 0.06 spine/μm in males; *p* = 0.53, unpaired *t* test; Figure 2C). Dendritic spine dynamics were explored in terms of turnover ratio (TOR) over 24 h, i.e., the combined fraction of dendritic spines that appears or disappears between consecutive imaging sessions, 24 h apart, to assess overall change. This measurement was obtained for each stage of the estrous cycle and showed no significant differences between stages (P: 0.13 ± 0.01; E: 0.13 ± 0.02; M/D: 0.12 ± 0.01; *p* = 0.63, repeated measures one-way ANOVA; Figure 2D). When the averaged TOR for female and male mice were compared, we did not find differences either (0.13 ± 0.01 in females vs. 0.16 ± 0.01 in males; *p* = 0.09, unpaired *t*-test; Figure 2E). To determine whether dendritic spines present or formed at a given stage of the estrous cycle were more stable than when this occurred at any of the other stages, we computed the survival function over a period of 6 days of two populations of spines: (1) all the dendritic spines present on the first occurrence of a given stage of the estrous cycle; and (2) new persistent spines formed on the first occurrence of each stage of the cycle. There were no differences in either the fraction of persistent spines (i.e., plateau; *p* = 0.6569, extra sum-of-squares *F* test) or rate constant K (*p* = 0.6621; extra sum-of-squares *F* test) between stages or comparable data from male mice (Figure 2F) when all the spines present at a given day were tracked. We did not find differences either for new persistent spines (*p* = 0.4721 and *p* = 0.4546 for fraction of persistent spines and K, respectively, extra sum-of-squares *F* test; Figure 2G). These data suggest that the stability of dendritic spines during steady-state conditions does not depend on the phase of the estrous cycle at which the spine was formed.

**Figure 3:**
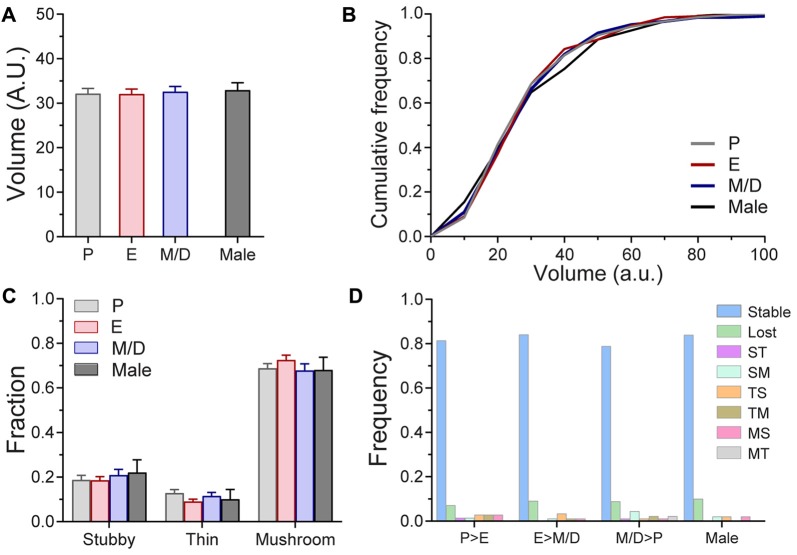
Volume of mushroom spines and morphology of dendritic spines of L5 pyramidal neurons in S1BF across the estrous cycle and male mice in steady-state conditions. **(A)** Volume of mushroom dendritic spines at the different stages of the estrous cycle and male mice. **(B)** Cumulative frequency distribution of the volume of mushroom spines at different stages of the estrous cycle and male mice. **(C)** Distribution of stubby, thin and mushroom spines at different stages of the estrous cycle and in male mice. **(D)** Frequency of spine type transitions between stages of the estrous cycle and in male mice. P, Proestrus; E, Estrus; M/D, Metestrus/Diestrus; ST, Stubby > Thin; SM, Stubby > Mushroom; TS, Thin > Stubby; TM, Thin > Mushroom; MS, Mushroom > Stubby; MT, Mushroom > Thin.

Next, we seek to elucidate whether oscillations in endogenous hormones, despite the fact that they do not influence the density or dynamics of dendritic spines, may still induce changes at the spine level, either by affecting the volume of mushroom spines or changing the proportions of the three main subtypes of dendritic spines. We used volumetric estimation and an unbiased semi-automated classification scheme (see “Materials and Methods” section) to analyze these parameters across the estrous cycle. We tracked and compared the volume of 210 individual spines across three consecutive stages of the estrous cycle and we did not find differences between stages (P: 32.12 ± 1.2; E: 32.07 ± 1.1; M/D: 32.57 ± 11.2; AU; *p* = 0.79, repeated measures one-way ANOVA; Figure 3A), or when the comparison included male mice (Males: 32.93 ± 1.6 AU; *p* = 0.9669, one-way ANOVA; Figure 3A). The medians and the 25% and 75% percentiles of the distributions of the volume of dendritic spines at different stages of the cycle and males were comparable (Medians: P: 27.64; E: 28.13; M/D: 29.66; Male: 30.6; 25% Percentiles: P: 20.63; E: 20.43; M/D: 20.08; Male: 18.49. Average: 19.91; and 75% Percentiles: P: 40.09; E: 39.99; M/D: 40.73; Male: 43.67; Figure 3B). Regarding the morphological subtypes of dendritic spines, analysis of the proportions of the presence of each subtypes at the population level indicated that there was no difference in the proportions of the stubby, thin, and mushroom spines between stages of the estrus cycle and male mice (*p* = 0.3754, *χ*^2^ test; Figure 3C). Taking advantage of the longitudinal aspect of our study we tracked the morphology of individual dendritic spines during the estrous cycle. Dendritic spines were tracked over the transitions from P > E, E > M/D, and from M/D > P. Depending on the morphology of the spine before and after those transitions, these were classified as: stubby > stubby (stable stubby), stubby > thin (ST), stubby > mushroom (SM), stubby > lost, thin > thin (stable thin), thin > stubby (TS), thin > mushroom (TM), thin > lost, mushroom > mushroom (stable mushroom), mushroom > stubby (MS), mushroom > thin (MT), and mushroom > lost. The fraction of stable spines (stable stubby, stable thin, and stable mushroom) was very similar for all the cycle transitions (P > E: 0.81 ± 0.07; E > M/D: 0.82 ± 0.1; M/D > P: 0.80 ± 0.12) as well as for males over a 24 h period (0.82 ± 0.08; *p* = 0.6614, one-way ANOVA), indicating that, regardless of the stage of the cycle or the sex of the animal, ~81% of the dendritic spines do not transition to other types of spines over a 24-h period (Figure 3D). The fraction of dendritic spines that were lost ranged between 0.071 and 0.088 in the females and it was 0.095 for males. The rest of the transitions (ST, SM, TS, TM, MS, and MT) presented very low frequencies (less than 0.04). Chi-square test did not find statistical differences between stages of the cycle or sex (*χ*^2^ *p* = 0.9017).

Despite the unique hormonal fluctuations of female mice our findings indicate that there are no observable changes in steady-state dendritic spine density, dynamics, survival, volume, or morphology in L5 pyramidal neurons of S1BF across the estrous cycle. Our results also indicate that spine density and dynamics in these neurons are comparable between female and male mice.

### Differential Effect of Sensory-Evoked Plasticity on the Dynamics of Dendritic Spines in S1BF L5 Pyramidal Neurons During the Estrous Cycle

**Figure 4:**
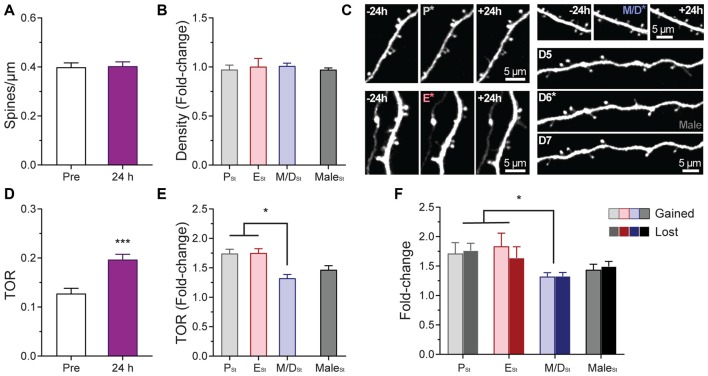
Effects of sensory-evoked plasticity on density and turnover of dendritic spines of L5 pyramidal neurons in S1BF at different stages of the estrous cycle. **(A)** Effect of rhythmic whisker stimulation on the density of dendritic spines when the data from the steady-state imaging sessions prior to the whisker stimulation were averaged to serve as a baseline (Pre vs. 24 h). **(B)** Change in the density of dendritic spines at 24 h based on the stage of the cycle at which the stimulation occurred. Data from male mice is also included in the analysis. **(C)** Representative *in vivo* two-photon images of apical dendritic segments from L5 pyramidal neurons taken 24 h apart depicting differences in relative changes of turnover ratio after whisker stimulation at different stages of the cycle and in male mice. Stimulation ocurred at P (top-left image sequence), E (bottom-left image sequence); M/D (top-right image sequence); and after the imaging session on the 6th day in male mice (bottom-right image sequence). Whisker stimulation was performed immediately following the 2PE imaging session depicted with an asterisk in the image. All are best projections (3–5 slices, 1.5 μm apart). **(D)** Effect of rhythmic whisker stimulation on the TOR of dendritic spines when the data from the steady-state imaging sessions prior to the whisker stimulation were averaged to serve as a baseline (Pre vs. 24 h). **(E)** Change in the TOR of dendritic spines at 24 h based on the stage of the cycle at which the stimulation occurred. **(F)** Changes in the gained and lost ratios based on the stage of the cycle at which the stimulation occurred. Data from male mice is also included in these two analyses. P_St_, E_St_, M/D_St_, and Male_St_: Stimulation at proestrus, estrus, metestrus/diestrus, and after the imaging session on 6th day in male mice, respectively. *p < 0.05 and ***p < 0.001.

While our results indicate that the estrous cycle does not have an effect on steady-state structural synaptic plasticity in L5 pyramidal neurons of S1BF, little is known about the effects of changing levels of endogenous hormones on experience-dependent synaptic plasticity in the cerebral cortex. Furthermore, no chronic *in vivo* studies have been performed to assess whether any particular stage of the estrous cycle favors synaptic plasticity or not. To answer this question, once the baseline imaging of dendritic spines during the estrous cycle was completed, females were randomly assigned to an experimental group based on the stage of the cycle (P_St_, E_St_, or M/D_St_) at which the mouse would undergo rhythmic whisker stimulation to induce sensory-evoked plasticity. Spine density and TOR of individual cells were compared pre- and post-stimulation. Data from the steady-state imaging sessions prior to the whisker stimulation were averaged to serve as a baseline (Pre) for comparison to the day after the whisker stimulation (24 h). Whisker stimulation did not affect density of dendritic spines either when pooling the data from the different stages together (Pre: 0.40 ± 0.06; 24 h: 0.40 ± 0.06; *p* = 0.72, paired *t* test; Figure 4A) or when comparisons were done as the fold-change in density based on the stage of the cycle of the mouse and the sex (*p* = 0.8451; Figure 4B). On the other hand, whisker stimulation increased the TOR of dendritic spines when combining the data from all the cells regardless of the stage at stimulation (PRE: 0.13 ± 0.03; 24 h: 0.20 ± 0.04; *p* < 0.001, paired *t test;* Figures 4C,D). Furthermore, when we divided the results based upon the stage of the cycle the female was in at the moment of the induction of plasticity, we found differential increases in TOR among stages. The fold-changes in TOR after the stimulation relative to the steady-state TOR were significantly larger when females were stimulated during P (P_St_: 1.74 ± 0.15) and E (E_St_: 1.75 ± 0.13) than when females were stimulated in the M/D stage (M/D_St_: 1.32 ± 0.13; *p* < 0.01 vs. P_St_ and E_St_, one-way ANOVA; Figures 3C,E). The fold-change in TOR found in males (1.47 ± 0.2) did not significantly differ from the values obtained for any of the female stages (Figure 4E). When we analyzed the independent contributions of the two factors that define TOR, we found that rates at which the values for gained and lost spines changed after the whisker stimulation were comparable at each estrous stage and in males (*p* = 0.8479, two-way ANOVA) whereas these values were different between stages and with males (*p* = 0.0074, two-way ANOVA; *p* < 0.05 for P_St_ vs. M/D_St_ and E_St_ vs. M/D_St_, Sidak’s multiple comparisons test; Figure 4F). Interestingly, when the data for fold-change in TOR were grouped based on the stage of the estrous cycle at which the female was in 24 h after the stimulation—when the *in vivo* imaging was performed—we failed to find any differences between stages (P_St_: 1.52 ± 0.26; E_St_: 1.73 ± 0.12; M/D_St_: 1.55 ± 0.31; *p* = 0.21, one-way ANOVA; not shown).

**Figure 5:**
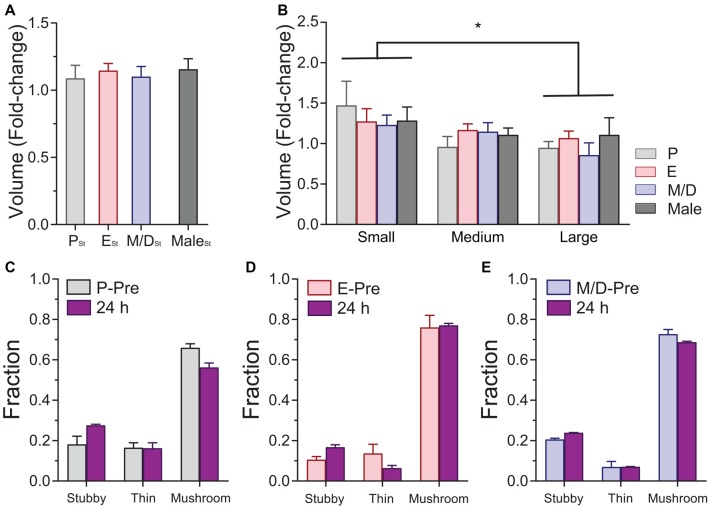
Effects of sensory-evoked plasticity on volume and morphology of dendritic spines of L5 pyramidal neurons in S1BF at different stages of the estrous cycle and in male mice. **(A)** Changes in the volume of mushroom spines at 24 h based on the stage of the cycle at which the stimulation occurred. **(B)** Differential effect of stimulation on the relative volume increase of dendritic spines. Small spines increase their volume relatively more than larger spines regardless of the stage of the cycle when the stimulation occurred or the sex. **(C–E)** Distribution of stubby, thin, and mushroom spines before (P-Pre, Proestrus Pre; E-Pre, Estrous Pre; and M/D-Pre, Metestrus/Diestrus Pre) and 24 h after sensory evoked plasticity at different stages of the estrous cycle. P_St_, E_St_, and M/D_St_: Stimulation at proestrus, estrus, and metestrus/diestrus, respectively. *p < 0.05.

Similarly to the analysis carried out during the *in vivo* imaging of steady-state plasticity, we studied the potential variations in volume in mushroom spines after the stimulation. Volume of individual dendritic spines were recorded before and after stimulation and the fold-change in volume was analyzed as a function of the stage of the cycle the female was in during the stimulation or sex. We could not find differences in spine volume fold-change between groups after stimulation (*p* = 0.9096, one-way ANOVA; Figure 5A). In an attempt to unmask if stimulation produces differential effects on spines depending on the spines size and if this effect was influenced by the stage of the estrous cycle the female was in at the time of the stimulation, we divided the population of spines of each experimental group in three size groups (small, medium, and large) based on the parameters of the distributions obtained during the steady-state conditions (averaged 25% percentile: 19.91; averaged 75% percentile: 41.12). Small spines were those with initial volume (volume measured at the imaging session immediately preceding the whisker stimulation) between 0 and 19.91 AU. Medium spines were those with initial volumes between 19.91 and 41.12 AU, and large spines those with a volume bigger than 41.12 AU. Data from male mice was divided in the same fashion. Analysis of the estrous stage/sex and spine size effects on the fold-change in volume was performed. The main effect of the factor size of the spines was statistically significant (*p* = 0.0116, two-way ANOVA; small vs. large, *p* = 0149, Tukey’s multiple comparisons test; Figure 5B), whereas there was no main effect due to the stage of the cycle or the sex or to the interaction size*estrous cycle/sex (*p* = 0.8042 and *p* = 0.7185, respectively, two-way ANOVA). These data indicate that smaller spines increase their relative size more than larger spines regardless of the stage of the cycle of the sex. Analysis of the proportions of the different types of dendritic spines at the population level showed no differences between estrous stages after the stimulation, regardless of the stage during the stimulation (*χ*^2^ *p* values: P_St_: 0.29; E_St_: 0.24; M/D_St_: 0.83; Figures 5C–E) or the stage 24 h after the stimulation (*χ*^2^ *p* values: P_St24_: 0.59; E_St24_: 0.16; M/D_St24_: 0.50; not shown). When we tracked the morphology of individual dendritic spines before and 24 h after the stimulation at different stages of the cycle, we did not find an effect of the stimulation on the frequencies of the transitions when comparing the frequencies obtained between stages of the cycle or sex (*p* = 0.9678, *χ*^2^ test; not shown).

Our results indicate that sensory-evoked plasticity does not result in changes in the density and morphology of dendritic spines at any stage of the estrous cycle in female mice, and comparable results were obtained in male mice. However, we find differential degrees of TOR of dendritic spines across the cycle after sensory stimulation. Small dendritic spines increase their relative size in response to sensory-evoked plasticity to a larger degree than large spines regardless of the stage of the cycle or the sex of the mouse.

## Discussion

The present *in vivo* imaging study finds that oscillatory levels of endogenous gonadal hormones during the estrous cycle do not have an effect on the steady-state density, dynamics, survival fraction, morphology, and volume of dendritic spines of L5 pyramidal neurons in S1BF. Our results suggest, however, that sensory-evoked synaptic plasticity may be differentially affected by the levels of gonadal hormones across the estrous cycle.

The estrous—or reproductive—cycle, characterized by cyclical alterations in the female reproductive track and in sexual receptivity, is regulated by the production and release of hormones by the brain, pituitary gland, and gonads. The effects of gonadal hormones on brain function have been widely described (Luine, 2008; Frick, 2009). In particular, the effects of exogenous estrogen on the hippocampus and cortex (Gould et al., 1990; Daniel et al., 1997; Woolley, 1998; Hao et al., 2006; Chen et al., 2009) suggest that these brain areas may be susceptible to changes in the levels of this hormone occurring during the different stages of the estrous cycle. In this regard, our results indicate that, similar to what has been reported for the visual cortex (Jeong et al., 2011), the somatosensory cortex of mice is equipped with the machinery to be sensitive to endogenous estrogens regardless of its source—i.e., brain-derived or circulating estrogen, and to the exposure to xenoestrogens like BPA (Kelly et al., 2014). However, very little is known about changes in the density or in the dynamics of dendritic spines in the cortex during endogenous oscillations of estrogens. The few studies examining the changes in density of dendritic spines in the cerebral cortex during the estrous cycle describe contradictory results, reporting either no differences across the cycle in the neocortex (Prange-Kiel et al., 2008) and in the prefrontal cortex of rats (Markham and Juraska, 2002), or higher density of dendritic spines during P in the primary somatosensory cortex of female rats (Chen et al., 2009). While these studies are comprehensive analyses of the density of dendritic spines throughout the estrous cycle, they lack the longitudinal aspect that *in vivo* studies provide. Our study following the same apical dendritic segments (within the first 100 mm from the pia mater) of L5 pyramidal neurons in S1BF during the estrous cycle (in several instances, up to three cycles) indicates that in the absence of significant sensory stimulation—limited to the social interaction with their cagemates and the exploration of the physical features of the home cage—the density of dendritic spines is stable during steady-state conditions with no significant changes across the different stages of the cycle. To the best of our knowledge this is the first comprehensive *in vivo* study on dendritic spine density, dynamics, survival fraction, size, and morphology done throughout the estrous cycle in normally cycling female mice. The fact that our study was done in young (2–5 months old) mice (most of the studies done in the cortex used rats) and limited to the apical dendrites of L5 pyramidal neurons has also to be taken into consideration when comparing these results with previously reported data. It is important to note that unaltered spine density does not rule out unaltered spine dynamics, as it has been previously observed (Trachtenberg et al., 2002; Keck et al., 2008). In fact, the rates at which dendritic spines are formed and eliminated could be different from day to day, not affecting, however, the overall density if both rates are equalized. These potential shifts are undetectable to fixed tissue studies. Our *in vivo* approach allowed us to test whether TOR varies from one stage of the cycle to another or stays constant throughout the cycle. We find that the TOR over 24 h does not change during the different stages of the estrous cycle suggesting that in steady-state conditions the naturally occurring oscillations in the levels of circulating estrogens do not influence the rate at which dendritic spines are formed and eliminated. We also find that these measurements, density and TOR, from cycling female mice are comparable to male mice, indicating that apical dendrites of L5 pyramidal neurons in S1BF of female and male mice have similar densities of dendritic spines and rewire cortical connections at similar rates. Another question we were able to answer was if dendritic spines formed at different stages of the cycle show varied degrees of spine stability. We analyzed the survival fraction of newly-formed spines that were formed at different phases of the cycle and lasted at least 24 h (new persistent spines). We found that this metric was not altered across the estrous cycle, suggesting that the oscillating levels of circulating estrogen during the cycle do not have an effect on the persistence of cortical synapses. The fact that spine morphology and volume of mushroom spines were unaltered during the estrous cycle reinforces the notion of a very stable, yet plastic, steady-state cortical micro-circuitry not altered by endogenous oscillations of gonadal hormones. The discrepancies with the previously reported results (Chen et al., 2009) may arise due to different subtypes of neurons examined. While in the present study we imaged non-adapting pyramidal track-type L5 pyramidal neurons exclusively (Hattox and Nelson, 2007; Popescu et al., 2017), there is no information about the subtype of L5 pyramidal neurons they examined. Furthermore, while our *in vivo* preparation is non-invasive, the *ex vivo* preparation described by Chen et al. (2009) requires slicing of the brain which severs almost all the horizontal projections onto these neurons and probably affects the natural occurrence of synaptic plasticity in the cortical circuits.

Rearrangement of the connections of neuronal circuits is necessary for synaptic plasticity, and hence for memory and learning. We wondered if changes in hormone levels during the estrous cycle may have a differential effect on the degree of rewiring of the L5 pyramidal neurons in S1BF after sensory-evoked synaptic plasticity by rhythmic whisker stimulation (Mégevand et al., 2009; Gambino et al., 2014). The plasticity-inducing protocol produced a generalized increase in TOR of dendritic spines, regardless of the stage of the cycle at which the female mice were stimulated. However, this increase in TOR was higher when the mice were stimulated during P and E than when the mice were stimulated during M/D, suggesting that indeed, some stages of the cycle (P and E) may favor the formation and elimination of synaptic connections, i.e., rewiring, to adapt to and integrate new information in the cortical microcircuits, while other stages (M and D) may favor the use of pre-existing connections. The fact that during steady-state conditions we did not find differences in TOR but we do after whisker stimulation is in agreement with the idea that gonadal hormones may serve as primers for synaptic plasticity, contingent to the co-occurrence of sustained synaptic activity within a time window (Srivastava and Penzes, 2011). In this sense, *in vitro* studies using cultured cortical neurons have shown rapid and transient increases in connectivity after treatment with estradiol (Srivastava et al., 2008). As the authors suggest, these results support the idea of a two-step model of estrogen-induced synaptic plasticity. In this view, formation of new non-functional dendritic protrusions is induced by elevated levels of estrogens leading some of them to form stable synapses after subsequent presynaptic stimulation, therefore contributing to remodeling of cortical circuits. The levels of gonadal estrogen at M and D are low and have been low for more than 24 h (Kato et al., 2013), probably outside of the sensitive window for plasticity elicited by the high levels of estrogen during P, and may explain the attenuated evoked-synaptic plasticity detected at M/D. Regarding the size and morphology of dendritic spines, we did not find differences in the volume or proportion of the three types of spines after stimulation, even when we sub-analyzed the results based on the stage of the cycle when the female was stimulated. It is important to note that while estrogens have been extensively studied and implicated in synaptogenesis and synaptic plasticity, the synergistic or antagonistic potential effects of the rest of the gonadal hormones should be considered when interpreting these results. In addition, the 24-h interval regime of our *in vivo* study may have missed events, e.g., transient spines, volume oscillations, etc. so we cannot draw any conclusion about differential immediate effects of evoked-plasticity during estrous cycle.

In summary, our findings indicate that steady-state density and dynamics of dendritic spines of L5 pyramidal neurons in the primary somatosensory cortex of female mice are constant during the estrous cycle; however, the susceptibility of these neurons to sensory-evoked structural plasticity is dependent on the current stage of the cycle. Since the dendritic spines of these neurons undergo higher degree of remodeling during proestrus and estrus than during metestrus/diestrus, it is possible that certain stages of the cycle could favor forms of memory requiring *de novo* formation and elimination of dendritic spines, i.e., rewiring of cortical circuits, while other stages are more suitable for forms of memory where retention or repurposing of already existing synaptic connections—or just changes is the strength of the synaptic contact—is more pertinent.

## Author Contributions

BHA, ET and RM performed the cranial window surgeries; BHA, HMB, ET and AMD collected the *in vivo* two-photon imaging data; AMD, BOO and SHL performed the ddPCR and the Western Blot experiments; HMB, ET, AMD, BOO, SHL and RM analyzed the data; HMB and ET contributed to the drafting and editing of the manuscript; BHA, AMD and RM contributed to the writing of the manuscript. All the authors contributed to the design of the experiments.

## Conflict of Interest Statement

The authors declare that the research was conducted in the absence of any commercial or financial relationships that could be construed as a potential conflict of interest.

## Footnotes

^1^http://rsb.info.nih.gov/ij/
